# Regenerative Potential of Adipose Tissue–Derived Exosomes in Treating Hair Shaft Abnormalities: A Case Report

**DOI:** 10.1155/crdm/8838962

**Published:** 2025-09-11

**Authors:** Mohammad Ali Nilforoushzadeh, Amirhossein Heidari, Nazila Heidari, Niloufar Najar Nobari

**Affiliations:** ^1^Skin Repair Research Center, Jordan Dermatology and Hair Transplantation Center, Shahid Beheshti University of Medical Sciences, Tehran, Iran; ^2^Skin and Stem Cell Research Center, Tehran University of Medical Sciences, Tehran, Iran; ^3^Faculty of Medicine, Tehran Medical Sciences, Islamic Azad University, Tehran, Iran; ^4^School of Medicine, Iran University of Medical Sciences, Tehran, Iran

**Keywords:** adipose tissue, case report, exosome, hair shaft anomaly

## Abstract

Hair shaft abnormalities, often linked to genetic or acquired conditions, present significant treatment challenges with limited and inconsistent therapeutic success. This report describes an eight-year-old female with severe hair shaft abnormality, unresponsive to standard treatments, including topical minoxidil and platelet-rich plasma therapy, who demonstrated marked improvement following a single session of adipose tissue (AT)–derived exosome therapy. The exosomes were administered via intradermal scalp injections, resulting in significant enhancement in hair structure, strength, and growth within 3 months, with no adverse effects observed. AT-derived exosomes, leveraging mechanisms such as Wnt/β-catenin activation and vascular endothelial growth factor expression, hold substantial promise for promoting hair follicle regeneration. This case highlights the potential of exosome therapy as an innovative and effective treatment for hair shaft abnormalities, emphasizing the need for further clinical studies to validate its efficacy and safety.

## 1. Introduction

While hair does not serve a vital function, it can be a marker of human health. Clinical and morphological abnormalities in hair can provide insights into particular complex diseases [1]. Hair is an ectodermal structure originating from the epidermal layer [2]. The hair shaft formation in humans is driven by a complicated interplay of biological signals within the pilosebaceous unit. Various cell types are recruited in the hair cycle to develop different parts of the hair, including the inner root sheath, cuticle, cortex, and medulla [3]. Cell differentiation in hair arises from the expression of major hair keratin genes, making keratins and keratin-associated proteins the primary biochemical components of hair. Changes in the aforementioned components following genetic alteration, as well as environmental triggers, modify the appearance, texture, growth, or manageability of the hair shaft, known as hair shaft abnormality.

Hair shaft abnormalities in children can manifest sporadically or following both acquired and genetic diseases [2]. No definitive treatment has been confirmed for this condition yet; however, control of the underlying condition and medications such as topical minoxidil, topical tretinoin, oral supplements, and energy-based approaches have been reported in some cases [4]. Exosomes, a class of extracellular vesicles, play a vital role in regulating paracrine signaling, and exosomes derived from dermal papilla cells may be essential for hair follicle regeneration through various mechanisms [5]. Herein, we described a case of hair shaft abnormality that favorably responded to the adipose tissue (AT)–derived exosome.

## 2. Case Presentation

**Figure 1:**
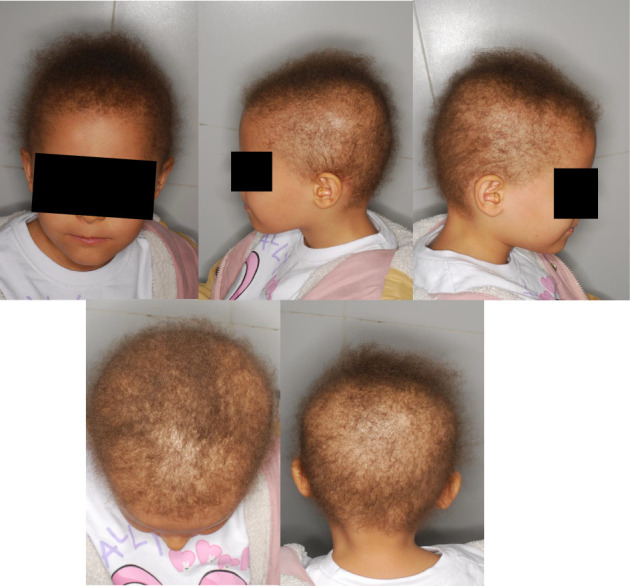
Clinical presentation of the patient before treatment with AT-derived exosomes. The scalp shows sparse, brittle, curly, and fragile hair with significant thinning and slow growth. The photograph highlights the key features of hair shaft abnormality, including diffuse hair loss and increased fragility (as confirmed by the tug test).

An eight-year-old female patient exhibited severe hair shaft abnormality accompanied by significant psychological distress. Upon admission, physical examination revealed diffuse, sparse, thinned, curly, slow-growing, and fragile hair over the scalp (Figure 1). The tug test was positive, confirming increased hair fragility. The patient mentioned a negative family history of hair abnormality. Her medical history was otherwise unremarkable for systemic diseases. Before admission to this center, the patient visited various dermatologists and underwent different therapeutic approaches, including topical minoxidil 5%, as well as platelet-rich plasma therapy. However, she failed all previous treatments, and her condition got worse over time. Recent preclinical and clinical studies have demonstrated that AT-derived exosomes can promote hair follicle regeneration through mechanisms such as activation of the Wnt/β-catenin pathway, stimulation of dermal papilla cell proliferation, and enhanced vascular endothelial growth factor (VEGF) expression [6]. Notably, the Wnt/β-catenin pathway is one of the signaling pathways that are involved in hair follicle morphogenesis and development [7]. These mechanisms align closely with the pathophysiology of hair shaft abnormalities, which involve defective hair formation and follicular miniaturization. Therefore, the patient was a candidate for nonautologous human AT-derived exosomes. The method of exosome harvesting from AT has been described in the previous investigation [8]. Before initiating exosome therapy, the patient underwent a thorough laboratory assessment, including a complete blood count with differential, comprehensive metabolic panel, fasting lipid profile, and screening for HIV, tuberculosis, and hepatitis B and C, all of which were within normal limits, confirming the absence of underlying systemic or infectious conditions. Moreover, the patient's parents obtained an informed consent form before the treatment initiation. AT-derived exosome was administered in one session, containing 2 mL of AT-derived exosome solution. The targeted area was prepared 30 min before the procedure by cleansing with sterile gauze and decontaminating with 70% alcohol pads. Afterward, local anesthesia was induced by injecting a 2% lidocaine solution to establish ring-block anesthesia. The procedure was subsequently performed by applying intradermal injections to the targeted areas of the scalp using a 1-cc syringe fitted with 27-gauge needles. The injection sites were spaced approximately 0.5 to 1 cm apart. The patient was then recommended to avoid scalp cleansing for the following 24 h. Standardized clinical photographs were obtained using a Nikon 10.2-megapixel camera at baseline and three months following treatment.

**Figure 2:**
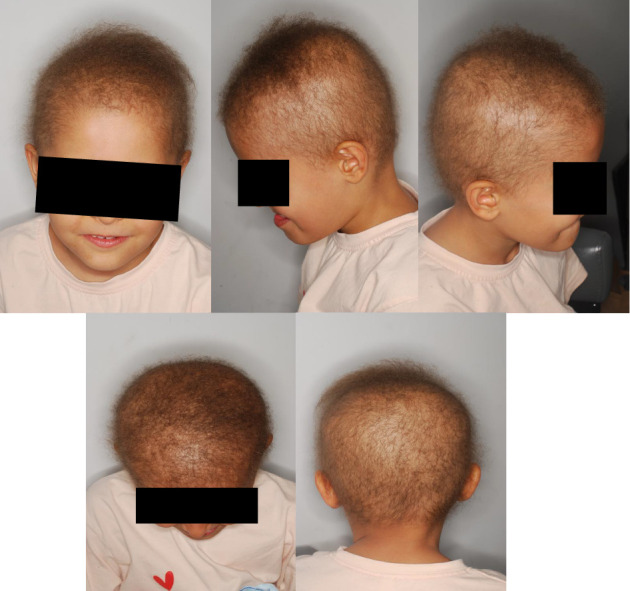
Improved hair structure and density 3 months after a single session of AT-derived exosome therapy. The photograph demonstrates enhanced hair quality, reduced brittleness, and better manageability, with no adverse reactions reported during the follow-up period.

The patient was revisited after three months following the exosome injection. Signs and symptoms were remarkably improved at the time of the visit, as dermatological evaluations showed increased hair density, improved fine hair texture, and darker hair color. The treatment was also safe, and no adverse reaction was reported. Additionally, the laboratory results remained within normal limits at the 3-month follow-up examination.  Figure 2 illustrates the clinical status at the 3-month follow-up. The patient is currently undergoing bimonthly evaluations, and extended follow-up at 6 and 12 months is planned to assess sustained clinical improvement. Additionally, depending on the progression of hair regeneration and patient response, further exosome therapy sessions may be considered.

## 3. Discussion

Regenerative medicine aims to restore or establish normal body function by substituting or regenerating cells, tissues, and organs. Previous investigations have demonstrated that mesenchymal stem cells (MSCs) could potentially promote hair formation through hair follicle remodeling [9]. Exosomes, nanovesicles ranging from 30 to 150 nm in diameter, provide several benefits over MSCs, including simplified storage and transportation, along with enhanced biosafety and reduced immunogenicity. Exosomes potentially contribute to cellular interaction, tissue homeostasis, cell differentiation, organogenesis, and tissue restructuring [10]. Moreover, exosomes can enhance tissue repair by carrying crucial factors, such as proteins, RNA, and microRNA.

Exosomes are currently utilized across various medical fields for their therapeutic, diagnostic, and drug-delivery capabilities [11]. In the field of dermatology, exosomes have been applied in both cosmetic and therapeutic areas, such as hypopigmented conditions, inflammatory disorders, wound healing, scar healing, aging, and hair growth [12, 13]. A combination of needle radiofrequency with topical exosomes has been correlated with satisfactory results in patients suffering from moderate to severe acne vulgaris [14]. Moreover, it has been proposed that exosomes, derived from both hair follicle regions and other tissue sources, can influence various components of hair follicles, including the dermal papilla, outer root sheath, and bulge [10].

Various preclinical studies have supported the potential of different tissue-derived exosomes in hair growth and rejuvenation. In a recent investigation, utilizing umbilical MSC-derived exosomes in a murine model of alopecia areata was correlated with enhanced hair follicle keratinocyte proliferation and hair regrowth [9]. Furthermore, exosomes derived from human AT and platelet-rich plasma potentially stimulate hair dermal papilla cell proliferation [15]. AT-derived exosomes enable interorgan communication by delivering to specific cells or tissues via paracrine or endocrine mechanisms [10]. Moreover, AT-derived exosomes proved to enhance hair regrowth by stimulating the proliferation of dermal papilla cells following the activation of the Wnt/β-catenin and TNF-α signaling pathways, along with elevating the expression of VEGF. All these findings highlighted the potential of exosomes as a therapeutic approach for conditions interrupting hair growth and rejuvenation.

There is no specific medication for hair shaft abnormalities. Based on the type of the disorder, various approaches have been utilized, including resolving the underlying condition, avoiding trauma and chemical products, and applying treatments such as topical minoxidil, photochemotherapy, laser therapy, and supplemental therapy [4]. In three cases of congenital hypotrichosis following ectodermal dysplasia, topical cetirizine solution (2 mL once daily) and oral vitamin D supplementation for a duration of 6 months led to an elevation in the density of hair on the scalp and replacement of vellus hair with terminal hair [16]. Notably, in a recent case series of three patients suffering from trichorrhexis nodosa, a type of hair shaft abnormality, four treatment sessions with MSC-derived exosomes resulted in a significant improvement in hair quality [17].

There is currently no definitive treatment for hair shaft abnormalities, so exosome therapy presents a promising potential avenue for patients suffering from these conditions. Nonetheless, further investigations with a larger sample size are required to validate the efficacy and safety of regenerative medicine in terms of hair rejuvenation in such disorders. Moreover, variations in dosing and administration regimens of exosome therapy present challenges in comparing outcomes across studies. Therefore, future studies should establish standardized protocols, including exosome concentration, volume per session, number of sessions, and injection intervals. Such standardization will be critical for ensuring consistency, optimizing efficacy, and facilitating reproducibility in clinical practice. Lastly, histopathological samples were not performed due to its invasiveness in pediatrics and clinical improvement.

In conclusion, despite the challenges posed by hair shaft abnormalities, current treatment options remain limited and often ineffective, emphasizing the critical need to develop novel therapeutic approaches. Exosome therapy, a novel emerging treatment, holds promise due to its ability to promote hair follicle regeneration and improve hair structure through various biological mechanisms. In our case, the application of AT-derived exosomes resulted in marked improvement in hair structure and strength, with no reported adverse effects, suggesting the potential for exosome therapy in hair regrowth. Further studies with larger sample sizes and longer follow-ups are warranted to establish the efficacy and safety of this treatment in hair loss conditions.

## Data Availability

The data that support the findings of this study are available from the corresponding author upon reasonable request.
